# A Continuous Adherence Score, but Not Dichotomous BAASIS Screening, Is Associated with Graft Function in Kidney Transplant Recipients

**DOI:** 10.3390/jcm15218227

**Published:** 2026-10-22

**Authors:** Justyna Zachciał, Izabela Uchmanowicz, Krzysztof Benc, Magdalena Kuriata-Kordek, Dariusz Janczak, Mirosław Banasik

**Affiliations:** 1 Department of Nursing, Faculty of Nursing and Midwifery, Wrocław Medical University, 51-618 Wroclaw, Poland; justyna.zachcial@umw.edu.pl (J.Z.); izabela.uchmanowicz@umw.edu.pl (I.U.); 2 Department and Clinic of Nephrology, Transplantation Medicine, and Internal Diseases, Wrocław Medical University, 50-556 Wroclaw, Poland; magdalena.kuriata-kordek@umw.edu.pl (M.K.-K.); miroslaw.banasik@umw.edu.pl (M.B.); 3 Department and Clinic of Vascular, General, and Transplantation Surgery, Wrocław Medical University, 50-556 Wroclaw, Poland; dariusz.janczak@umw.edu.pl

**Keywords:** kidney transplantation, medication adherence, BAASIS, ACDS, acceptance of illness, eGFR, graft function, psychosocial factors, cross-sectional study

## Abstract

**Background/Objectives**: Non-adherence to immunosuppressive medication is a principal modifiable determinant of kidney allograft survival, yet its associations with objective graft function remain inconsistent, in part because the Basel Assessment of Adherence to Immunosuppressive Medications Scale (BAASIS) dichotomises infrequent and persistent non-adherence together. We tested whether a continuous adherence measure and psychological acceptance of illness are more closely associated with graft function than BAASIS. **Methods**: Cross-sectional, single-centre study of 230 adult kidney transplant recipients; the primary analysis included 199 recipients with eGFR measured on the day of assessment and complete covariate data. Approximately 95% of recipients had received a deceased-donor and 5% a living-donor graft. Adherence was measured by BAASIS, the Adherence in Chronic Diseases Scale (ACDS), and the Adherence to Refills and Medications Scale (ARMS). Psychosocial covariates included the Acceptance of Illness Scale (AIS), Hospital Anxiety and Depression Scale—Modified (HADS-M), WHO Quality of Life—BREF (WHOQOL-BREF), Tilburg Frailty Indicator (TFI), and Mini-Mental State Examination (MMSE). The primary outcome was eGFR on the day of assessment; secondary and exploratory outcomes were serum creatinine and the individual eGFR slope reconstructed from annual archival records (*n* = 147). Multivariable linear regression adjusted for age, sex, time since transplantation, and psychosocial covariates was complemented by a linear mixed-effects model (158 recipients, 747 eGFR observations) and by sensitivity analyses, including adjustment for the immunosuppressive regimen. **Results**: Among 199 recipients (46.2% female; mean age 58.5 ± 14.0 years; median time since transplantation 6.5 years; calcineurin inhibitor in 95.2% of those with regimen data), 36 (18.1%) met the BAASIS criterion for non-adherence, most frequently due to timing deviation > 2 h. BAASIS non-adherence was not associated with eGFR (*p* = 0.14) or eGFR slope (*p* = 0.23). ACDS correlated with eGFR (ρ = +0.22, *p* = 0.002) and was independently associated with higher eGFR (β = +1.78 mL/min/1.73 m^2^ per ACDS point, 95% CI +0.56 to +3.00; *p* = 0.005; adjusted R^2^ = 0.083) and with lower log-creatinine (β = −0.051; *p* = 0.025). Mean eGFR across ACDS tertiles was 35.9, 40.5, and 44.6 mL/min/1.73 m^2^ (Kruskal–Wallis *p* = 0.034). The association was robust to adjustment for immunosuppressive regimen and to removal of the least consistent ACDS item. The mixed-effects model showed an average eGFR decline of 1.21 mL/min/1.73 m^2^ per year (*p* < 0.001), with no significant adherence × time or psychosocial × time interactions. Acceptance of illness was not independently associated with eGFR (*p* = 0.17). **Conclusions**: In kidney transplant recipients, dichotomous BAASIS screening was not associated with graft function, whereas higher continuous ACDS scores were independently associated with higher eGFR. Given the cross-sectional design, the low internal consistency of the ACDS in this sample, and the modest explained variance, these findings are hypothesis-generating and warrant prospective confirmation with objective adherence measures.

## 1. Introduction

Kidney transplantation (KTx) remains the renal replacement therapy of choice for most patients with end-stage kidney disease, offering superior long-term survival and health-related quality of life compared with maintenance dialysis. Progressive decline in graft function over time, however, remains common, and the rate of post-transplant eGFR decline has been directly linked to graft loss and patient mortality [1]. Characterising the trajectory of kidney function over time has therefore become a central endpoint in nephrology research [2], with increasingly sophisticated approaches to modelling multi-phasic decline after transplantation [3,4] and in native kidney disease [5,6,7]. Against this background, identifying modifiable determinants of graft function, and of its trajectory over time, is a priority for post-transplant care.

Among modifiable determinants, adherence to immunosuppressive medication is arguably the most important. Non-adherence has been associated with allograft rejection, chronic graft dysfunction, and graft loss across multiple cohorts [8,9,10], and a recent prospective multi-assessment study confirmed that self-reported BAASIS non-adherence independently predicted biopsy-proven rejection [11]. Yet reported prevalences of non-adherence in KTx recipients vary very widely, from approximately 2% to 78%, depending on the assessment method, population, and time since transplantation [10,12]. This heterogeneity reflects both the intrinsic difficulty of measuring medication-taking behaviour and the multifactorial nature of adherence, which integrates regimen complexity, beliefs about medication, psychological functioning, cognitive status, and biological factors such as frailty and comorbid burden [13,14,15].

The BAASIS has become the de facto reference self-report instrument in transplantation research. It assesses four implementation dimensions, dose taking, drug holidays, timing deviations > 2 h, and dose reduction, together with an item on discontinuation, classifying any affirmative response as non-adherent [9,11]. Although BAASIS is brief, validated, and widely used, its dichotomous output has been criticised for limited sensitivity: patients with occasional lapses are grouped together with those who take large proportions of prescribed doses incorrectly, which may dilute associations with clinical endpoints [12]. Complementary self-report instruments offer continuous quantification. The Adherence in Chronic Diseases Scale (ACDS) is a seven-item instrument developed and validated in Polish patients with chronic conditions, yielding a score from 0 to 28 where higher values indicate better adherence [16]; the Adherence to Refills and Medications Scale (ARMS) provides a complementary twelve-item estimate across taking and refilling behaviour [17].

Beyond adherence itself, psychological adaptation to chronic disease, operationalised through the Acceptance of Illness Scale [18], and emotional state, cognitive status, frailty, and health-related quality of life [19,20,21,22] are plausible determinants of medication-taking behaviour and may themselves be directly associated with graft function. Longitudinal work in Chinese KTx recipients has shown, for example, that lower early post-transplant eGFR is associated with higher subsequent depressive symptoms [23], and trajectory-based analyses increasingly support the view that psychosocial profiles themselves follow distinct temporal patterns after transplantation [24,25].

In a prior analysis of a KTx cohort at our centre [26], psychosocial and age-related factors including anxiety, cognitive functions, age, and time since transplantation were independently associated with BAASIS adherence. A companion analysis from the same group focused on the psychosocial determinants of the ARMS score [27]. Neither study, however, incorporated clinical graft-function data, and neither could therefore test whether psychosocial drivers of non-adherence translate into measurable differences in kidney function. The present study uses the same centre population, linked with eGFR measured on the day of assessment and with archival post-transplant laboratory data, to address this gap, and to explicitly compare the clinical discriminative performance of dichotomous BAASIS with continuous ACDS and ARMS.

## 2. Materials and Methods

### 2.1. Study Design and Setting

This was a cross-sectional, single-centre study with prospective enrolment conducted at the Department and Clinic of Nephrology, Transplantation Medicine, and Internal Diseases, Wrocław Medical University, Poland. Between 28 December 2018 and 22 June 2022, adult KTx recipients attending routine outpatient follow-up were consecutively invited to participate. Enrolment consisted of informing the patient about the study, obtaining written informed consent, and administering the questionnaires during a routine visit; no study-specific interventions or additional procedures were performed. Graft-function data were subsequently extracted from the electronic medical records (Section 2.3.3), and the analysis was performed after data collection was complete. The study protocol was approved by the local Bioethics Committee of Wrocław Medical University (approval No. 789/2018, 27 December 2018; the approval was granted before the first participant was enrolled and covered the entire data-collection period) and was conducted in accordance with the Declaration of Helsinki. All participants provided written informed consent before enrolment.

### 2.2. Participants and Analysis Samples

Of 230 recipients enrolled and assessed with the psychosocial battery, the primary cross-sectional analysis included the 199 recipients (86.5%) with eGFR recorded on the day of the questionnaire assessment and complete data on all covariates; 31 recipients were excluded because eGFR on the day of assessment was not recorded (*n* = 28) or because the transplantation date or BAASIS data were missing (*n* = 3). The longitudinal analyses required archival post-transplant eGFR values: the individual eGFR slope was estimated in 147 recipients with at least two annual post-transplant values, and the linear mixed-effects model included 158 recipients with at least one value and complete covariates; 66 enrolled recipients, predominantly those transplanted many years before the assessment, had no annual post-transplant eGFR in the archival records. A comparison of included versus excluded participants is reported in Table A1 (Appendix A). Approximately 95% of the recipients had received a kidney from a deceased donor and 5% from a living donor; donor type was not recorded in the research database at the individual level.

Inclusion criteria were age ≥ 18 years, functioning renal allograft at the time of assessment, at least 3 weeks since transplantation, and ability to complete self-report questionnaires in Polish. Exclusion criteria were acute medical instability, severe cognitive impairment precluding valid self-report, and refusal of consent.

### 2.3. Measures

#### 2.3.1. Adherence

**BAASIS**: Five items on missed doses, drug holidays (≥2 consecutive missed days), timing deviation > 2 h, dose reduction without medical advice, and discontinuation within the preceding four weeks [9]. A “yes” to any item classified the participant as non-adherent. Item 5 additionally elicited a self-reported adherence percentage (0–100%).

**ACDS**: Seven items scored 0–4, total 0–28; higher = better adherence [16].

**ARMS**: Twelve items scored 1–4, total 12–48; higher = worse adherence [17].

#### 2.3.2. Psychosocial Instruments

**AIS**: Eight items scored 1–5, total 8–40; higher = greater acceptance of illness [18].

**HADS-M**: Sixteen items: standard 14-item HADS (7-item anxiety and 7-item depression subscales; [19]) augmented with a two-item irritability subscale. Items 2, 4, 6, 7, 12, and 14 were reverse-scored per the standard HADS algorithm; each subscale range is 0–21.

**WHOQOL-BREF**: Twenty-six items yielding four domain scores, physical, psychological, social relationships, and environment, transformed to a 4–20 scale [20].

**TFI**: Fifteen-item Part B assessing physical, psychological, and social frailty [22], scored according to the original scoring instructions (total 0–15; higher = greater frailty). Item-level coding was verified against the original questionnaires.

**MMSE**: Standard 30-point cognitive screening [21]. Cognitive impairment was operationalised as MMSE < 27.

#### 2.3.3. Clinical Data

Serum creatinine and eGFR (CKD-EPI; [28]) were extracted from the electronic medical record at approximately 3 weeks post-KTx (discharge) and annually thereafter up to 10 years; eGFR was additionally recorded on the day of the questionnaire assessment. The primary outcome was eGFR on the day of assessment, which is temporally aligned with the adherence and psychosocial measurements. Because serum creatinine was not recorded on the day of assessment, the most recent annual archival creatinine value was used as a secondary outcome. Individual eGFR slope (mL/min/1.73 m^2^/year) was estimated by ordinary least-squares regression of post-transplant eGFR on year, requiring at least two post-transplant eGFR measurements; this approach is consistent with established eGFR trajectory reconstruction from archival clinical records [3,4,29]. GFR was estimated rather than determined as creatinine clearance, because 24 h urine collections were not performed systematically during outpatient follow-up, are prone to collection error, and overestimate GFR owing to tubular creatinine secretion; current guidance suggests estimating GFR from serum creatinine whenever creatinine is measured in kidney transplant recipients [30]. The current immunosuppressive regimen was coded from the medication list recorded during the BAASIS interview into binary indicators for tacrolimus (immediate- or prolonged-release), cyclosporine A, mycophenolic acid derivatives, corticosteroids, mTOR inhibitors, and azathioprine. Tacrolimus trough concentrations were monitored at every outpatient visit as part of routine care but were not included in the research database.

### 2.4. Statistical Analysis

Continuous variables are presented as mean ± SD or median [IQR], and categorical variables as *n* (%). Normality was assessed with the Shapiro–Wilk test; because most variables were not normally distributed, bivariate comparisons used the Mann–Whitney U, Kruskal–Wallis, or Fisher’s exact test, and bivariate associations were examined with Spearman rank correlation, which makes no distributional assumption. The primary analysis, defined in line with the study objective, was the association of ACDS with current graft function in multivariable ordinary least-squares regression, with eGFR on the day of assessment as the primary outcome (*n* = 199) and log-transformed serum creatinine as the secondary outcome (*n* = 147); Holm correction was applied to these two tests. Models included age, sex, time since transplantation, ACDS, AIS, HADS-M anxiety, HADS-M depression, and BAASIS dichotomous status. The distributional assumptions of the linear models concern the residuals, which were inspected graphically, and multicollinearity was assessed with variance inflation factors (VIF). All other analyses, including the eGFR slope model, were considered exploratory.

Because the OLS-estimated individual eGFR slope relies on fitting a straight line through two to ten archival values per recipient and is sensitive to uneven measurement density, a recognised limitation of reconstructed trajectory analyses [2,7], a longitudinal analysis was performed using a linear mixed-effects model (LMM) with a Gaussian distribution and identity link on long-format data including all recipients with at least one post-transplant eGFR value and complete covariates (747 observations across 158 recipients); likelihood-based estimation provides valid estimates under the missing-at-random assumption without requiring complete follow-up. Models included a fixed linear effect of time (years post-transplant), the same fixed covariates as in the cross-sectional analysis, and subject-specific random intercepts, with or without random slopes. Nested models were compared by likelihood-ratio tests using maximum likelihood estimation, supplemented by the Akaike and Bayesian information criteria (AIC, BIC); for the random slope, the *p*-value was obtained from a 50:50 mixture of χ^2^(1) and χ^2^(2) distributions. Year × ACDS, year × BAASIS, and year × AIS interactions were added in separate models to test whether adherence or illness acceptance modifies the rate of eGFR decline. The final model was re-estimated by restricted maximum likelihood, and marginal and conditional R^2^ were calculated according to Nakagawa and Schielzeth [31]. The complete-case model (147 recipients) was estimated as a sensitivity analysis.

Sensitivity analyses of the primary model included additional adjustment for immunosuppressive regimen (tacrolimus, cyclosporine A, corticosteroid), exclusion of ACDS item 6 (the item with the lowest item–total correlation), item-level models, and the outcome definition used in the originally submitted analysis (most recent annual archival eGFR). Sensitivity analyses of the slope model included additional adjustment for the number of eGFR measurements and the duration of follow-up, and restriction to recipients with at least three measurements. The reliability of each self-report instrument was assessed with Cronbach’s α (Table 1 footer), and the item-level performance of the ACDS was examined (Supplementary Table S1). Analyses used Python 3.12 (pandas, SciPy, statsmodels); statistical code was prepared with the assistance of a generative AI tool (Claude Opus 5.5, Anthropic) and verified by the authors. Two-sided *p* < 0.05 was considered statistically significant. Exploratory analyses are reported without adjustment for multiple testing, and their *p*-values should be interpreted as descriptive.

## 3. Results

### 3.1. Sample Characteristics

The primary analysis sample comprised 199 KTx recipients (92 women, 46.2%) with a mean age of 58.5 ± 14.0 years. Median time since transplantation was 6.5 years (IQR 2.0–14.7). Approximately 95% of recipients had received a deceased-donor kidney and 5% a living-donor kidney. Among the 187 recipients with regimen data, 178 (95.2%) were treated with a calcineurin inhibitor (tacrolimus 70.6%, cyclosporine A 24.6%), 97 (51.9%) with a mycophenolic acid derivative, and 69 (36.9%) with oral corticosteroids. Baseline characteristics and psychosocial scores are reported in Table 1. Table A1 (Appendix A) compares included participants (*n* = 199) with those excluded (*n* = 31); excluded participants were somewhat older (median 67.0 vs. 63.0 years, *p* = 0.050) and further from transplantation (median 14.4 vs. 6.5 years, *p* = 0.003), but did not differ in adherence, psychosocial scores, cognitive function, or frailty.

### 3.2. Adherence Prevalence and BAASIS Versus Graft Function

Thirty-six participants (18.1%) met the BAASIS criterion for non-adherence. Timing deviation > 2 h was the most prevalent single behaviour (31 patients; 15.6%), followed by missed doses (17; 8.5%); other forms of non-adherence were infrequent (≤4%). Adherent and non-adherent recipients (by BAASIS) did not differ in eGFR on the day of assessment (median 40.0 vs. 30.0 mL/min/1.73 m^2^, *p* = 0.140), current creatinine (1.75 vs. 1.50 mg/dL, *p* = 0.910; *n* = 147), or eGFR slope (−1.0 vs. −1.3 mL/min/1.73 m^2^/year, *p* = 0.229; *n* = 147). ARMS (median 14 vs. 16, *p* < 0.001) and ACDS (26 vs. 25, *p* = 0.042) differed in the expected direction between BAASIS groups, supporting convergence between adherence measures.

### 3.3. Spearman Correlations

Table 2 presents Spearman correlations between covariates and graft function (exploratory). ACDS correlated positively with eGFR on the day of assessment (ρ = +0.222, *p* = 0.002) and inversely with current creatinine (ρ = −0.197, *p* = 0.017), but not with eGFR slope. Time since transplantation correlated inversely with eGFR (ρ = −0.170, *p* = 0.017). AIS showed a non-significant trend with creatinine (ρ = −0.146, *p* = 0.077). BAASIS self-reported percentage, ARMS, HADS-M, WHOQOL-BREF, TFI, and MMSE showed no significant associations with any outcome. Age correlated with eGFR slope (ρ = +0.241, *p* = 0.003), with older recipients showing less steep decline, and inversely with creatinine (ρ = −0.209, *p* = 0.011).

### 3.4. Multivariable Analysis

In the primary multivariable model of eGFR on the day of assessment (*n* = 199; R^2^ = 0.120; adjusted R^2^ = 0.083; F = 3.24, *p* = 0.002), higher ACDS was independently associated with higher eGFR (β = +1.78 mL/min/1.73 m^2^ per ACDS point, 95% CI +0.56 to +3.00; *p* = 0.005), and longer time since transplantation with lower eGFR (β = −0.48 per year, *p* = 0.021); age showed a borderline association (*p* = 0.050). BAASIS dichotomous status, sex, AIS, and HADS-M subscales were not independently associated with eGFR (Table 3). VIFs ranged from 1.06 to 2.08, indicating no relevant multicollinearity. The adjusted R^2^ indicates that the model explains a modest proportion of the variance in eGFR. Specifically, the full model explained 12.0% of the variance in eGFR (8.3% after adjustment for the number of covariates), of which ACDS uniquely accounted for approximately 4% (partial R^2^ = 0.042).

In the secondary model of log-creatinine (*n* = 147; adjusted R^2^ = 0.149; F *p* < 0.001), higher ACDS was independently associated with lower log-creatinine (β = −0.051 per point, 95% CI −0.095 to −0.006; *p* = 0.025), as were higher AIS (β = −0.015, *p* = 0.030), older age (*p* = 0.005), and female sex (*p* = 0.020). After Holm correction for the two primary tests, the ACDS associations remained statistically significant (adjusted *p* = 0.009 for eGFR and *p* = 0.025 for log-creatinine).

In the exploratory model of eGFR slope (*n* = 147; adjusted R^2^ = 0.113; F *p* = 0.002), older age (β = +0.096 per year, 95% CI +0.007 to +0.185; *p* = 0.035), higher AIS (β = +0.197, *p* = 0.038), and female sex (β = +2.83, *p* = 0.034) were associated with less steep decline, whereas ACDS (*p* = 0.090) and BAASIS status (*p* = 0.284) were not. The association of age with slope persisted after additional adjustment for the number of eGFR measurements and the duration of follow-up (β = +0.091, *p* = 0.047) and was stronger in recipients with at least three measurements (*n* = 129; β = +0.148, 95% CI +0.075 to +0.221; *p* < 0.001).

### 3.5. ACDS Tertile Analysis

**Figure 1:**
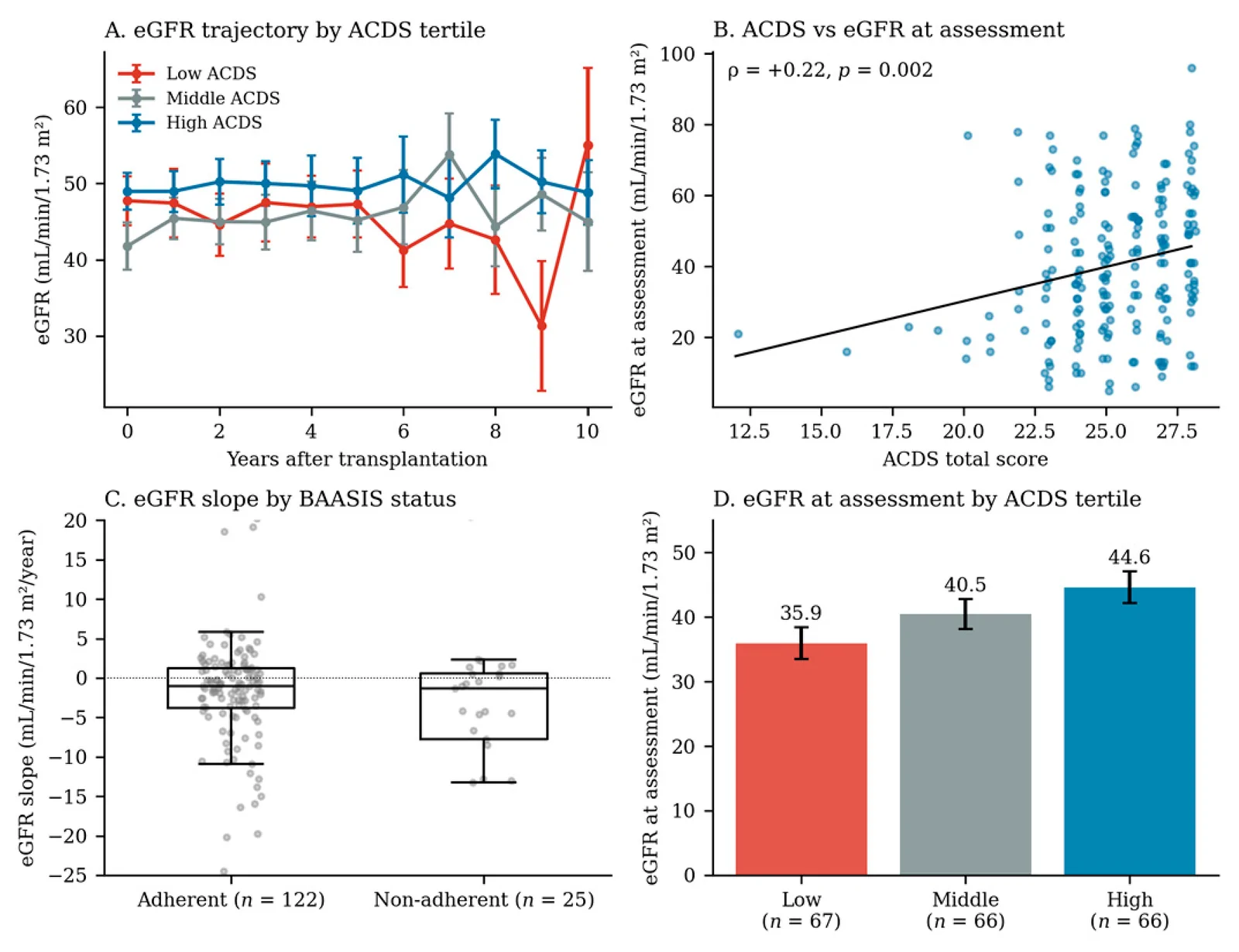
Graft function by adherence measure. (**A**) Mean post-transplant eGFR trajectory stratified by ACDS tertile (*n* = 147), with standard-error bars; year-points displayed where *n* ≥ 5. (**B**) ACDS score versus eGFR on the day of assessment (*n* = 199), with least-squares line. (**C**) eGFR slope by BAASIS status (*n* = 147), no significant difference (Mann–Whitney *p* = 0.23); *y*-axis truncated at −25 and +20 mL/min/1.73 m^2^/year. (**D**) eGFR on the day of assessment by ACDS tertile (*n* = 199; mean ± standard error; Kruskal–Wallis *p* = 0.034).

To illustrate the magnitude of the association, participants were stratified into ACDS tertiles by rank. Mean eGFR on the day of assessment was 35.9 ± 20.1 mL/min/1.73 m^2^ in the lowest tertile (*n* = 67), 40.5 ± 18.7 in the middle tertile (*n* = 66), and 44.6 ± 19.9 in the highest tertile (*n* = 66), a monotonic gradient of approximately 9 mL/min/1.73 m^2^ (Kruskal–Wallis *p* = 0.034). Figure 1 illustrates these patterns and the absence of differentiation between BAASIS-adherent and BAASIS-non-adherent groups.

### 3.6. Longitudinal Analysis by Linear Mixed-Effects Model

The linear mixed-effects model included 747 post-transplant eGFR observations across 158 recipients (mean 4.7 measurements per recipient; range 1–10). Random slopes substantially improved model fit compared with random intercepts only (likelihood-ratio χ^2^ = 45.7, *p* < 0.001; ΔAIC = −41.7), whereas adding the year × ACDS (χ^2^ = 3.46, *p* = 0.063; ΔAIC = −1.5; ΔBIC = +3.2), year × BAASIS (*p* = 0.484), or year × AIS interaction (*p* = 0.626) did not significantly improve fit (Supplementary Table S2). In the final model with random intercepts and slopes, the mean fixed effect of time was β = −1.21 mL/min/1.73 m^2^ per year (95% CI −1.85 to −0.57; *p* < 0.001), confirming ongoing eGFR decline in the cohort. ACDS was associated with a higher eGFR level across follow-up (β = +1.53 per point, 95% CI +0.15 to +2.91; *p* = 0.030), whereas BAASIS status (*p* = 0.302) and AIS (*p* = 0.139) were not. The marginal and conditional R^2^ were 0.088 and 0.765, respectively, indicating that most of the variance in eGFR is attributable to stable between-recipient differences. Residuals were symmetric (skewness 0.01) with moderately heavy tails (excess kurtosis 4.4; Supplementary Figure S1). The complete-case model (147 recipients, 736 observations) gave consistent results (year β = −1.33; year × ACDS *p* = 0.073).

### 3.7. Sensitivity Analyses

The association between ACDS and eGFR on the day of assessment was robust across sensitivity analyses (Supplementary Table S3). Additional adjustment for tacrolimus, cyclosporine A, and corticosteroid use (*n* = 187) left the association essentially unchanged (β = +1.75, 95% CI +0.46 to +3.04; *p* = 0.008), and none of the regimen indicators was independently associated with eGFR (all *p* > 0.5). After exclusion of ACDS item 6, which asks whether the physician enquires about treatment-related inconvenience rather than assessing the patient’s own medication-taking behaviour and was practically unrelated to the remaining items (corrected item–total correlation 0.06), internal consistency increased to α = 0.53 and the association was slightly stronger (β = +2.31, 95% CI +0.64 to +3.98; *p* = 0.007). In item-level models, the association was driven mainly by items 4 (response to adverse drug effects; β = +5.74, *p* = 0.031) and 5 (perceived necessity of the prescribed medication; β = +4.59, *p* = 0.005), with all remaining items being directionally consistent (Supplementary Table S1). With the outcome definition used in the originally submitted analysis (most recent annual archival eGFR, *n* = 147), which was frequently recorded after the questionnaire assessment, the association was directionally consistent but not statistically significant (β = +1.55, 95% CI −0.10 to +3.20; *p* = 0.066). When the published ACDS adherence categories were applied (low ≤ 20, medium 21–26, high ≥ 27 points; *n* = 7, 123, and 69), mean eGFR increased across categories (27.4, 39.0, and 43.9 mL/min/1.73 m^2^; Kruskal–Wallis *p* = 0.042), but the adjusted difference between high and lower adherence did not reach statistical significance (β = +4.97, 95% CI −0.96 to +10.90; *p* = 0.100), consistent with the loss of information associated with dichotomising a continuous score.

## 4. Discussion

In this cross-sectional analysis of 199 KTx recipients with comprehensive psychosocial phenotyping, eGFR measured on the day of assessment, and retrospectively reconstructed eGFR trajectories, two findings stand out. First, dichotomous BAASIS non-adherence was not associated with eGFR, creatinine, or eGFR slope. Second, the continuous ACDS adherence score was independently associated with eGFR after adjustment for age, sex, time since transplantation, and psychosocial covariates, with a gradient of approximately 9 mL/min/1.73 m^2^ between the lowest and highest ACDS tertiles. Given the modest explained variance and the low internal consistency of the ACDS in our sample, this association should be regarded as modest in magnitude and hypothesis-generating. In particular, the adjusted R^2^ of 0.083 indicates that the ACDS association is relevant at the population level and does not support the use of ACDS for individual prediction of graft function.

The BAASIS non-adherence prevalence of 18.1% in our sample aligns with the lower end of the range reported in KTx populations (2.4–78%; [10,12]) and is lower than the 33.6% reported by Scheel et al. [14] in a German KTx cohort and the 55% cumulative prevalence recently reported in repeated-measures assessments [11]. Timing deviation > 2 h was the dominant non-adherent behaviour in our cohort, mirroring the finding of Haupenthal et al. [11] that non-adherence is primarily driven by punctuality problems and single missed doses. The apparent absence of a BAASIS-related signal in clinical endpoints in our cross-sectional sample is reconcilable with this literature: recent prospective work shows that BAASIS predicts rejection when used longitudinally with repeated assessments [11], but a single cross-sectional BAASIS, as used in most adherence-to-outcome studies, including ours, may have limited sensitivity because the dichotomous criterion groups patients with infrequent timing lapses together with those with persistent non-adherence [12]. Continuous measures such as ACDS, which quantify both frequency and commitment to the regimen, may therefore better approximate the cumulative adherence burden relevant to graft function in cross-sectional research.

The independent association of ACDS with graft function has, to our knowledge, not been previously demonstrated in KTx recipients with adjustment for BAASIS status. The direction of the approximately 9 mL/min/1.73 m^2^ eGFR difference between ACDS tertiles is consistent with estimates of the association between adherence and graft function from electronic-monitoring and tacrolimus-level-variability studies [8], although the cross-sectional design does not allow causal interpretation. Contrary to our initial hypothesis, acceptance of illness was not independently associated with eGFR (*p* = 0.17); it was associated with lower creatinine and less steep eGFR decline only in exploratory analyses. These exploratory findings are nonetheless compatible with a growing literature linking psychological adaptation to chronic disease with objective health outcomes, including evidence that early post-transplant renal function itself predicts subsequent depressive symptoms [23] and that kidney recipients follow distinct longitudinal psychological-distress trajectories after transplantation [24].

The positive association between recipient age and eGFR slope, with older recipients showing slower decline, is a recognised pattern in KTx and CKD cohorts [1,29] but should be interpreted with caution. First, it may partly reflect survivor bias: inclusion required a functioning graft at the time of assessment, so recipients whose rapid decline led to graft loss were not represented, and in older recipients death with a functioning graft is a frequent competing event that may remove patients before decline is observed. Second, the number and timing of eGFR measurements varied between recipients (2–10 per patient), and OLS slopes based on few measurements are imprecise and susceptible to regression to the mean; however, the association persisted after adjustment for the number of measurements and the duration of follow-up and was stronger in recipients with at least three measurements. Third, older recipients frequently receive kidneys from older donors with lower initial function, which limits the absolute decline that can be observed, whereas younger recipients have higher baseline eGFR and have been reported to show higher rates of acute rejection, chronic allograft injury, and non-adherence. Our linear mixed-effects model confirmed an average eGFR decline of 1.21 mL/min/1.73 m^2^ per year, a rate consistent with multi-phasic decline models reported in larger KTx cohorts [3,4], and did not detect a significant modifying effect of adherence or psychosocial variables on trajectory, although the year × ACDS interaction showed a non-significant trend (*p* = 0.063, likelihood-ratio test). The absence of a detectable adherence–slope association contrasts with some electronic-monitoring studies and likely reflects limited statistical power combined with modest between-patient variability in a stable outpatient cohort; group-based trajectory methods applied to larger KTx populations have identified distinct sub-phenotypes of eGFR decline [25] that OLS and LMM approaches are not designed to capture. Methodological work on longitudinal eGFR modelling [7,32] has further highlighted that different estimators can yield different effect-size estimates for the same covariate, and our use of both OLS slope and LMM provides convergent results.

A sensitivity analysis additionally adjusting for the immunosuppressive regimen left the ACDS–eGFR association essentially unchanged (β = +1.75 mL/min/1.73 m^2^ per ACDS point; *p* = 0.008). The ACDS association is therefore unlikely to be confounded by calcineurin-inhibitor class or corticosteroid exposure, although residual confounding by drug exposure, which could not be assessed without drug concentrations, cannot be excluded.

Our study extends the previous analyses of this centre’s KTx population [26,27] by integrating clinical graft-function data and by explicitly comparing adherence instruments head-to-head. The finding that a continuous Polish-validated adherence measure [16] was more closely associated with eGFR and creatinine than BAASIS suggests that a complementary continuous adherence assessment alongside BAASIS screening deserves prospective evaluation in clinical practice.

### 4.1. Potential Clinical Implications

A single BAASIS yes/no output may under-estimate clinically relevant adherence variation in routine post-transplant follow-up. If confirmed prospectively, incorporating continuous adherence instruments could help identify recipients whose graft function is lower than that of their peers and who may benefit from targeted pharmacist-led education, psychological support, or simplified regimens [33]. Our findings complement the recommendation of Haupenthal et al. [11] that BAASIS be integrated into routine post-transplant care by suggesting, as a hypothesis for prospective testing, that dichotomous screening may be more informative when combined with repeated assessment or with a parallel continuous instrument than when used as a single-time-point risk-stratification tool. At present, our findings do not support any change in clinical practice.

### 4.2. Limitations

Several limitations should be acknowledged. First, the analysis samples were subject to selection: 31 of 230 enrolled recipients (13.5%) could not be included in the primary analysis, mainly because eGFR on the day of assessment was not recorded, and the longitudinal analyses were restricted to 147–158 recipients with archival post-transplant eGFR data. Excluded recipients were older and further from transplantation, and the longitudinal samples over-represent recipients with shorter post-transplant follow-up, which limits generalisability to long-term recipients; although likelihood-based mixed models are valid under the missing-at-random assumption, missingness not at random cannot be excluded. Second, the cross-sectional design precludes causal inference; the association between ACDS and graft function could be bidirectional, for example, deteriorating graft function may itself erode adherence through increased treatment burden or emotional distress [23]. Third, adherence was assessed exclusively by self-report. Tacrolimus trough concentrations, although routinely monitored, were not available in the research database, so self-reported adherence could not be validated against drug levels or tacrolimus intra-patient variability, an established objective marker of non-adherence; no electronic medication-event monitoring was available; and prospective repeated BAASIS assessment, which has recently been shown to be necessary for optimal predictive validity [11], was not possible in this design. Fourth, the internal consistency of the ACDS was low in our sample (α = 0.36), reflecting a pronounced ceiling effect in six of the seven items and one item (item 6), which concerns the physician’s enquiry about treatment-related inconvenience rather than the patient’s own behaviour and was practically unrelated to the others; although the association with eGFR was preserved, and slightly stronger, after removal of this item, and random measurement error tends to bias associations towards the null, scale-based inferences should be treated as preliminary. The internal consistency of the WHOQOL-BREF physical domain was also low. Fifth, the explained variance was modest (adjusted R^2^ 0.083; marginal R^2^ of the mixed model 0.088), because graft function is determined by numerous donor, immunological, and peri-transplant factors that were not part of the present analysis; the ACDS association therefore has limited value for individual-level prediction. An explained variance of this magnitude is common in models relating a single behavioural factor to a multifactorial clinical outcome, and the finding should be interpreted as a modest population-level association rather than as a determinant of graft function in individual patients. In addition, the definition of the primary outcome was changed during revision: in the originally submitted analysis, current eGFR was defined as the most recent annual archival value, which was frequently recorded after the questionnaire assessment, whereas the revised primary outcome is eGFR measured on the day of assessment, which is temporally aligned with the questionnaires. With the originally submitted definition, the ACDS association was directionally consistent but not statistically significant (β = +1.55, 95% CI −0.10 to +3.20; *p* = 0.066). Donor type could not be included as a covariate because it was not recorded in the research database at the individual level; because only approximately 5% of recipients had received a living-donor graft, confounding by donor type is likely to be limited, although it cannot be excluded, nor can confounding by other donor characteristics such as donor age. Sixth, apart from the two primary tests, analyses were exploratory and not adjusted for multiple comparisons, which increases the risk of type I error. Seventh, eGFR slope was reconstructed retrospectively from archival records with variable follow-up density, reconstructed-trajectory methods have known limitations of their own [7,34], and serum creatinine was not recorded on the day of assessment, so the secondary creatinine outcome is not temporally aligned with the questionnaires. Eighth, hard clinical endpoints, graft loss and death, were not analysable in the source database at the time of data lock; linking adherence and psychosocial profile to these endpoints in an updated follow-up would be a natural next step, given that eGFR decline predicts hard outcomes in KTx [1]. Finally, the sample size was modest and derived from a single centre. With *n* = 199, the primary analysis had 80% power (two-sided α = 0.05) to detect correlations of |ρ| ≥ 0.20 and a covariate explaining ≥ 3.8% of residual variance; because only 36 recipients were classified as BAASIS non-adherent, the BAASIS comparison could reliably detect only moderate differences in graft function (Cohen’s d ≥ 0.52), and the null BAASIS findings should be interpreted as absence of evidence rather than evidence of absence. A larger cohort would allow for analysis of individual BAASIS dimensions, increase power to detect adherence × time interactions, and permit stratified analyses by donor type, time since transplantation, and immunosuppressive regimen; conversely, effect estimates obtained in modest samples tend to be inflated, and the ACDS association may be attenuated in larger cohorts. Validation in a larger, preferably multicentre cohort is planned. Strengths include comprehensive psychosocial phenotyping, multi-instrument adherence assessment, a primary outcome measured concurrently with the questionnaires, and linkage to routinely collected laboratory endpoints with both OLS and LMM trajectory reconstruction.

## 5. Conclusions

In this cross-sectional analysis of adult KTx recipients, dichotomous BAASIS screening was not associated with eGFR or the rate of eGFR decline, whereas higher continuous ACDS scores were independently associated with higher eGFR. Given the cross-sectional design, the possibility of reverse causality, the low internal consistency of the ACDS in this sample, and the modest explained variance, these findings should be regarded as hypothesis-generating. Prospective studies using objective adherence measures are needed to determine whether continuous adherence assessment adds clinically useful information to BAASIS screening.

## Figures and Tables

**Table 1:** Characteristics of the study sample (*n* = 199).

Summary	Variable
***Demographics***	
58.5 ± 14.0	Age, years (mean ± SD)
92 (46.2)	Female sex, *n* (%)
6.5 [2.0–14.7]	Time since KTx, years (median [IQR])
≈95/≈5	Donor type: deceased/living, %
***Adherence***	
36 (18.1)	BAASIS non-adherent, *n* (%)
31 (15.6)	Timing deviation > 2 h
17 (8.5)	Missed dose
8 (4.0)	Drug holiday (≥2 consecutive)
2 (1.0)	Dose reduction
2 (1.0)	Discontinuation
97.8 ± 6.6	BAASIS self-reported adherence, %
25.2 ± 2.4	ACDS total (0–28)
14.4 ± 2.5	ARMS total (12–48)
***Psychosocial***	
30.2 ± 8.0	AIS total (8–40)
5.4 ± 3.7	HADS-M anxiety (0–21)
3.7 ± 3.4	HADS-M depression (0–21)
12.9 ± 1.7	WHOQOL physical (4–20)
14.1 ± 2.0	WHOQOL psychological (4–20)
15.7 ± 2.7	WHOQOL social (4–20)
15.3 ± 2.1	WHOQOL environment (4–20)
27.1 ± 2.7	MMSE total (0–30)
75 (37.7)	MMSE < 27, *n* (%)
5.3 ± 2.8	TFI total (0–15)
***Immunosuppression (n = 187 with regimen data)***	
178 (95.2)	Calcineurin inhibitor, *n* (%)
132 (70.6)	Tacrolimus
46 (24.6)	Cyclosporine A
97 (51.9)	Mycophenolic acid derivative, *n* (%)
69 (36.9)	Oral corticosteroid, *n* (%)
7 (3.7)/5 (2.7)	mTOR inhibitor/azathioprine, *n* (%)
***Graft function***	
40.3 ± 19.8	eGFR on day of assessment, mL/min/1.73 m^2^
1.74 [1.25–2.99]	Current creatinine, mg/dL (*n* = 147), median [IQR]
−2.50 ± 8.04	eGFR slope, mL/min/1.73 m^2^/year (*n* = 147)

Values are mean ± SD unless stated otherwise. Scale internal consistency (Cronbach’s α): BAASIS 0.68; ACDS 0.36 (0.53 without item 6; Supplementary Table S1); ARMS 0.62; AIS 0.88; HADS-M anxiety 0.80, depression 0.81; WHOQOL-BREF physical 0.21, psychological 0.62, social 0.75, environment 0.75; TFI 0.70. The low ACDS α reflects a pronounced ceiling effect and one weakly related item; the low physical-domain WHOQOL α reflects item heterogeneity in this cohort. See Section 4.2, Limitations.

**Table 2:** Spearman correlations (ρ) between covariates and graft function (exploratory analysis).

eGFR Slope (*n* = 147)	Creatinine (*n* = 147)	eGFR at Assessment (*n* = 199)	Covariate
+0.241 **	−0.209 *	0.094	Age
0.022	0.004	−0.170 *	Time since KTx
0.105	−0.197 *	+0.222 **	ACDS total
−0.086	0.055	−0.015	ARMS total
0.117	−0.092	0.1	BAASIS self-reported %
0.1	−0.146	0.107	AIS total
0.072	0.026	−0.114	HADS-M anxiety
0.024	0.026	−0.034	HADS-M depression
0.026	−0.067	0.103	WHOQOL physical
−0.009	−0.088	−0.000	WHOQOL psychological
−0.074	0.056	−0.116	WHOQOL social
0.012	−0.013	0.056	WHOQOL environment
−0.100	0.084	−0.036	TFI total
0.045	−0.010	0.014	MMSE total

* *p* < 0.05; ** *p* < 0.01. Creatinine: most recent annual archival value.

**Table 3:** Multivariable linear regression, primary analysis (outcome: eGFR on the day of assessment, *n* = 199).

*p*	95% CI	β	Covariate
0.238	−58.17 to +14.51	−21.83	Constant
0.050	0.000 to +0.407	0.204	Age (years)
0.702	−4.426 to +6.560	1.067	Female sex
0.021 *	−0.887 to −0.074	−0.481	Time since KTx (years)
0.005 **	+0.558 to +3.001	1.779	ACDS total
0.170	−0.125 to +0.704	0.29	AIS total
0.192	−1.594 to +0.322	−0.636	HADS-M anxiety
0.070	−0.087 to +2.171	1.042	HADS-M depression
0.613	−9.005 to +5.326	−1.840	BAASIS non-adherent (yes)

Outcome: eGFR on the day of assessment (mL/min/1.73 m^2^). R^2^ = 0.120; adjusted R^2^ = 0.083; F = 3.24, *p* = 0.002. VIF 1.06–2.08. * *p* < 0.05; ** *p* < 0.01.

## Data Availability

De-identified data supporting the findings of this study are available from the corresponding author upon reasonable request.

## Supplementary Materials

The following supporting information can be downloaded at: https://www.mdpi.com/article/10.3390/jcm15218227/s1, Table S1: Item-level performance of the ACDS; Table S2: Linear mixed-effects model comparison; Table S3: Sensitivity analyses for the association of ACDS with eGFR; Figure S1: Residual diagnostics of the linear mixed-effects model.

## Abbreviations

The following abbreviations are used in this manuscript:

KTx	Kidney transplantation
BAASIS	Basel Assessment of Adherence to Immunosuppressive Medications Scale
ACDS	Adherence in Chronic Diseases Scale
ARMS	Adherence to Refills and Medications Scale
AIS	Acceptance of Illness Scale
HADS-M	Hospital Anxiety and Depression Scale—Modified
WHOQOL-BREF	World Health Organization Quality of Life—BREF
TFI	Tilburg Frailty Indicator
MMSE	Mini-Mental State Examination
eGFR	estimated glomerular filtration rate
CKD-EPI	Chronic Kidney Disease Epidemiology Collaboration (equation)
OLS	ordinary least squares
LMM	linear mixed-effects model
IQR	interquartile range
SD	standard deviation
CI	confidence interval

## Appendix A

**Table A1:** Comparison of recipients included in the primary analysis (*n* = 199) versus those excluded (*n* = 31).

*p*	Excluded (*n* = 31)	Included (*n* = 199)	Variable
0.050	67.0 [60.2–71.0]	63.0 [49.0–68.5]	Age, median [IQR] (years)
0.702	13 (41.9)	92 (46.2)	Female sex, *n* (%)
0.003	14.4 [9.6–16.7]	6.5 [2.0–14.7]	Time since KTx, median [IQR] (years)
1.000	5 (16.1)	36 (18.1)	BAASIS non-adherent, *n* (%)
0.163	26.0	25.0	ACDS total, median
0.408	34.0	31.0	AIS total, median
0.138	4.0	5.0	HADS-M anxiety, median
0.079	2.0	3.0	HADS-M depression, median
0.479	28.0	28.0	MMSE total, median
0.386	13.1	13.1	WHOQOL physical, median
0.648	5.0	5.0	TFI total, median

*p*-values from Mann–Whitney U test (continuous) or Fisher’s exact test (categorical). Reasons for exclusion: eGFR on the day of assessment not recorded (*n* = 28); missing transplantation date or BAASIS data (*n* = 3). Excluded recipients were older and further from transplantation but did not differ in adherence, psychosocial scores, cognitive function, or frailty.
