# Supplementary material for: A Continuous Adherence Score, but Not Dichotomous BAASIS Screening, Is Associated with Graft Function in Kidney Transplant Recipients
Source: J Clin Med. 2026 Oct 22;15(21):8227. doi: 10.3390/jcm15218227 (PMC13763649; doi:10.3390/jcm15218227)
Supplement: Supplementary file 1 [file jcm-15-08227-s001.zip › jcm-4484652-supplementary.pdf]

Supplementary Materials

Table S1. Item-level performance of the Adherence in Chronic Diseases Scale (ACDS) in the primary analysis sample (n = 199).

Item	Mean ± SD	At maximum, %	Corrected item–total r	α if item deleted	β for eGFR (95% CI)	p
1	3.79 ± 0.49	81	0.15	0.34	+0.37 (−5.39 to +6.14)	0.898
2	3.87 ± 0.40	89	0.28	0.30	+1.29 (−5.74 to +8.33)	0.717
3	3.92 ± 0.39	95	0.27	0.31	+5.37 (−1.70 to +12.45)	0.136
4	3.85 ± 0.54	91	0.21	0.31	+5.74 (+0.54 to +10.94)	0.031
5	3.53 ± 0.90	70	0.27	0.25	+4.59 (+1.39 to +7.78)	0.005
6	2.34 ± 1.43	28	0.06	0.53	+1.32 (−0.62 to +3.26)	0.181
7	3.92 ± 0.35	94	0.24	0.32	+0.94 (−7.34 to +9.23)	0.822

Cronbach's α of the full scale = 0.36; without item 6 = 0.53. Item-level models: each item replaced the ACDS total score in the primary multivariable model (outcome: eGFR on the day of assessment; adjusted for age, sex, time since transplantation, AIS, HADS-M anxiety, HADS-M depression, and BAASIS status). Items are scored 0–4, higher = better adherence. Item content: 1, remembering to take all medicines; 2, changing doses without consulting the physician; 3, adjusting medication to well-being; 4, response to adverse drug effects; 5, perceived necessity of the prescribed medication; 6, physician enquiring about treatment-related inconvenience; 7, honesty in reporting medication-taking to the physician.

Table S2. Comparison of linear mixed-effects models for post-transplant eGFR (158 recipients, 747 observations; maximum likelihood estimation).

Model	Log-likelihood	AIC	BIC	Comparison
M0: random intercept	−3070.8	6163.6	6214.3	—
M1: random intercept and slope (final)	−3047.9	6121.9	6181.9	vs M0: $\chi^2 = 45.7$, $p < 0.001$ *
M2: M1 + year × ACDS	−3046.2	6120.4	6185.1	vs M1: $\chi^2 = 3.46$, $p = 0.063$
M3: M1 + year × BAASIS	−3047.7	6123.4	6188.0	vs M1: $\chi^2 = 0.49$, $p = 0.484$
M4: M1 + year × AIS	−3047.8	6123.7	6188.3	vs M1: $\chi^2 = 0.24$, $p = 0.626$

*All models include fixed effects of year, age, sex, time since transplantation, ACDS, AIS, HADS-M anxiety, HADS-M depression, and BAASIS status. * p-value from a 50:50 mixture of $\chi^2(1)$ and $\chi^2(2)$ distributions (variance parameter on the boundary). Final model (M1, REML): year β = −1.21 (95% CI −1.85 to −0.57), p < 0.001; ACDS β = +1.53 (+0.15 to +2.91), p = 0.030; BAASIS β = +3.99 (−3.58 to +11.56), p = 0.302; AIS β = +0.32 (−0.10 to +0.74), p = 0.139. Marginal $R^2 = 0.088$; conditional $R^2 = 0.765$. Complete-case sensitivity model (147 recipients, 736 observations): year β = −1.33 (−2.02 to −0.63); year × ACDS β = +0.30, p = 0.073.*

Table S3. Sensitivity analyses for the association of ACDS with eGFR.

Analysis	n	ACDS β (95% CI)	p
Primary model	199	+1.78 (+0.56 to +3.00)	0.005
ACDS without item 6	199	+2.31 (+0.64 to +3.98)	0.007
Additional adjustment for tacrolimus, cyclosporine A, corticosteroid	187	+1.75 (+0.46 to +3.04)	0.008
Outcome: most recent annual archival eGFR (original definition)	147	+1.55 (−0.10 to +3.20)	0.066
Mixed-effects model, eGFR level across follow-up	158	+1.53 (+0.15 to +2.91)	0.030

β: difference in eGFR (mL/min/1.73 m^2) per one-point higher ACDS score, adjusted for age, sex, time since transplantation, AIS, HADS-M anxiety, HADS-M depression, and BAASIS status. None of the regimen indicators was independently associated with eGFR (all p > 0.5).

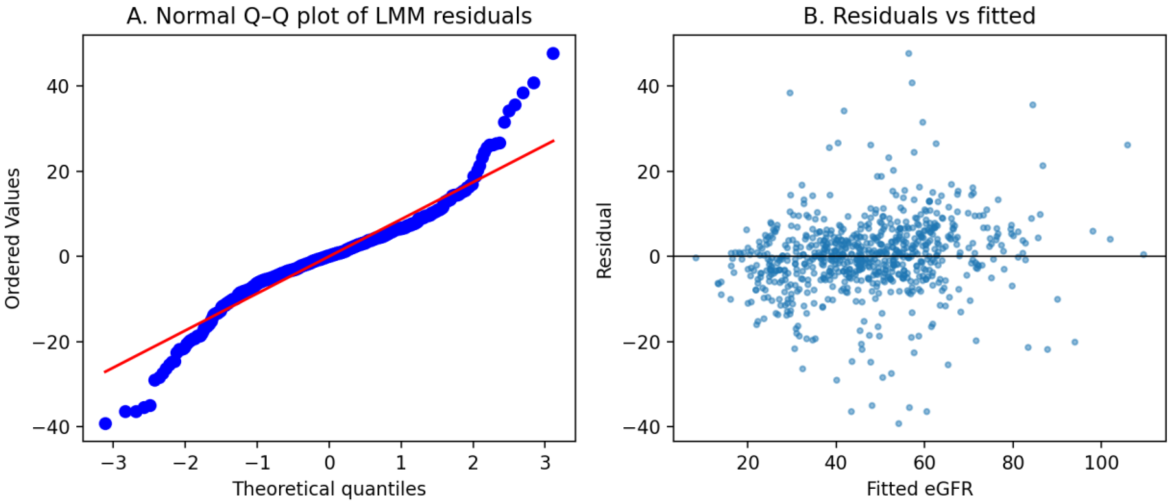

Figure S1. Residual diagnostics of the final linear mixed-effects model (158 recipients, 747 observations). (A) Normal Q–Q plot of residuals. (B) Residuals versus fitted values. Residuals were symmetric (skewness 0.01) with moderately heavy tails (excess kurtosis 4.4).
